# Extending the Range of Detectable Trace Species with the Fast Polarity Switching of Chemical Ionization Orbitrap Mass Spectrometry

**DOI:** 10.1021/acs.analchem.4c00650

**Published:** 2024-05-01

**Authors:** Runlong Cai, Joona Mikkilä, Anna Bengs, Mrisha Koirala, Jyri Mikkilä, Sebastian Holm, Paxton Juuti, Melissa Meder, Fariba Partovi, Aleksei Shcherbinin, Douglas Worsnop, Mikael Ehn, Juha Kangasluoma

**Affiliations:** † Institute for Atmospheric and Earth System Research/Physics, Faculty of Science, University of Helsinki, 00014 Helsinki, Finland; ‡ Shanghai Key Laboratory of Atmospheric Particle Pollution and Prevention (LAP3), Department of Environmental Science & Engineering, Fudan University, 200438 Shanghai, China; § Karsa Ltd., A. I. Virtasen aukio 1, 00560 Helsinki, Finland; ∥ Faculty of Engineering and Natural Sciences, Tampere University, 33720 Tampere, Finland; ⊥ Aerodyne Research, Inc., 45 Manning Road, Billerica, Massachusetts 01821, United States

## Abstract

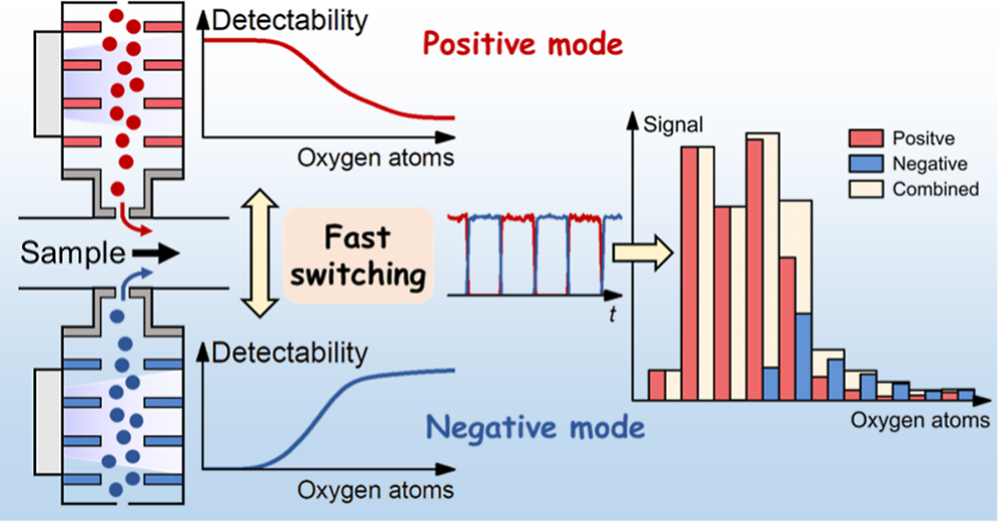

Chemical ionization (CI) atmospheric pressure interface mass spectrometry is a unique analytical technique for its low detection limits, softness to preserve molecular information, and selectivity for particular classes of species. Here, we present a fast polarity switching approach for highly sensitive online analysis of a wide range of trace species in complex samples using selective CI chemistries and high-resolution mass spectrometry. It is achieved by successfully coupling a multischeme chemical ionization inlet (MION) and an Orbitrap Fourier transform mass spectrometer. The capability to flexibly combine ionization chemistries from both polarities effectively extends the detectability compared to using only one ionization chemistry, as commonly used positive and negative reagent ions tend to be sensitive to different classes of species. We tested the performance of the MION-Orbitrap using reactive gaseous organic species generated by α-pinene ozonolysis in an environmental chamber and a standard mixture of 71 pesticides. Diethylammonium and nitrate are used as reagent ions in positive and negative polarities. We show that with a mass resolving power of 280,000, the MION-Orbitrap can switch and measure both polarities within 1 min, which is sufficiently fast and stable to follow the temporal evolution of reactive organic species and the thermal desorption profile of pesticides. We detected 23 of the 71 pesticides in the mixture using only nitrate as the reagent ion. Facilitated by polarity switching, we also detected 47 pesticides using diethylammonium, improving the total number of detected species to 59. For reactive organic species generated by α-pinene ozonolysis, we show that combining diethylammonium and nitrate addresses the need to measure oxygenated molecules in atmospheric environments with a wide range of oxidation states. These results indicate that the polarity switching MION-Orbitrap can promisingly serve as a versatile tool for the nontargeted chemical analysis of trace species in various applications.

## Introduction

Chemical ionization (CI) mass spectrometry is a versatile technique to analyze gaseous species with high selectivity and sensitivity.^1^ With the proper selection of ionization chemistry, a chemical ionization mass spectrometer (CIMS) can readily detect species of special interest and provide largely preserved molecular information. Integrating selective ionization chemistry at atmospheric pressure and high-resolution mass spectrometry has successfully addressed the specific needs for low detection limits,^2^ enabling direct online detection of trace species in complex gaseous samples with concentrations down to 10^4^ molecules cm^–3^. These advances have led to revolutions in, e.g., atmospheric chemistry in the past decade.^3,4^ Coupled with inlets that can vaporize sample molecules, CIMS can also be used to analyze liquid/solid samples such as explosives,^5,6^ pesticide residues,^7^ and aerosol particles.^8,9^

High chemical selectivity is fundamental to ionizing trace species of particular interest in a complex sample, yet by definition, it limits the concurrent ionization of species with a large variety in complex samples. For example, nitrate-CIMS has been widely used in atmospheric measurements for its high sensitivity and selectivity to gaseous sulfuric acid and highly oxygenated organic molecules (OOMs),^2,10^ which drive the formation and growth of secondary aerosol particles.^11,12^ However, the sensitivity of nitrate-CIMS tends to deteriorate as the oxidation state of the analyte decreases. Consequently, it misses a large fraction of reactive species that participate in atmospheric chemistry.^13^

Simultaneous measurements using multiple complementary ionization chemistries can address the need to analyze a wide variety of trace species. In addition to nitrate, other reagent ions such as iodide,^14^ bromide,^15^ acetate,^16^ trifluoromethoxide,^17^ hydronium,^18^ ammonium,^19^ aminium,^20^ protonated ethanol,^21^ etc., have been shown to be sensitive to certain classes of species. Compared to synchronizing multiple mass spectrometers, using one switchable CI inlet for multiple ionization chemistries with the same mass spectrometer has significant advantages in versatility, expense, and convenience of deployment. It also avoids the potential inconsistencies that come with the use of mass spectrometers with different sensitivities. Previous studies have shown that using a switchable CI inlet with nitrate and bromide can improve the comprehensiveness of the detected organic species.^22,23^

The ability to switch the polarity of CIMS can considerably extend the range of detectable species, as positive and negative ionization chemistries tend to complement each other.^24^ For instance, the proton transfer reaction is sensitive to reactive organic species with low oxidation states, while nitrate ionization is sensitive to highly oxidized species.^25^ This advantage in combining ionization chemistries in different polarities over the same polarity strongly motivates the development of a new generation of polarity switching CIMS for the online detection of trace species. Besides, polarity switching can suppress the interference among ionization chemistries caused by the inevitable diffusion of neutral reagent molecules, which benefits stability and robustness in long-term measurements. For instance, nitric acid used as the negative ion source is expected to have a minor influence on the positive mode because of its comparatively low proton affinity, which is also supported by our experimental results.

Among the existing CI inlets with the capability to switch ionization chemistries,^26,27^ the multischeme chemical ionization inlet (MION) offers a unique solution. It works at atmospheric pressure and accordingly avoids dilution of samples, which leads to the loss of high sensitivity. Further, it uses a purge flow in the opposite direction of ion trajectories to prevent neutral reagent molecules from entering the sampling line.^28^ This design can minimize the mixing of samples with electrically neutral reagent molecules. Thus, the MION can significantly reduce the interference among different ionization chemistries due to the “memory effect” of reagent molecules^22^ compared to switching neutral reactant molecules that enter the ionization region.

Sufficient switching frequency is vital for a polarity switching CIMS to follow the variation of analytes, and high resolving power is necessary for resolving their molecular formulas unambiguously. The Orbitrap Fourier transform mass spectrometer can switch polarity in ∼1 s,^29^ and its high resolving power (>100,000) over conventional online time-of-flight mass spectrometry (∼10,000) facilitates the identification of trace species in complex samples. This high resolving power can be particularly important for the nontargeted analysis of reactive organic species, as the analysis is usually challenging due to the high complexity of the spectrum. Coupling Orbitrap MS and CI at atmospheric pressure, the CI-Orbitrap has proven to be a powerful technique to analyze gaseous organic species,^30^ and its sensitivity to trace species has been improved for online analysis in atmospheric chemistry.^31^

Here, we present a polarity switching MION-Orbitrap, which can achieve the concurrent ionization and the detection of a wide range of trace species in complex samples by fast switching of the ionization chemistries between polarities (e.g., 1 min for each polarity). Diethylammonium (C_4_H_12_N^+^) and nitrate (NO_3_^–^) are used as reagent ions in positive and negative modes, respectively. The polarity switching MION-Orbitrap is tested with reactive organic species generated in an environmental chamber and pesticides vaporized by thermal desorption. The selectivity and sensitivity of C_4_H_12_N^+^ and NO_3_^–^ to these analytes are compared and discussed. Based on these experiments, we show that the polarity switching MION-Orbitrap is a promising new-generation technique to achieve fast online analysis of a wide range of trace species.

## Experimental Section

We evaluated the performance of a polarity switching MION-Orbitrap instrument using a standard pesticide sample and gaseous organic species generated by α-pinene ozonolysis. We used the latest version MION (MION2, Karsa Ltd.), which has an increased reagent ion concentration compared to previous versions and enabled the use of multiple CI chemistries with the same ionization time.^28^ The Orbitrap MS was a research-grade Q Exactive Plus Orbitrap MS^32,33^ (Thermo Fisher Scientific Inc.). We set its mass resolution to 280,000 (at *m*/*Q* = 200 Th) for resolving analytes in complex samples, though decreasing the mass resolution may facilitate faster polarity switching. The sensitivity of this Orbitrap MS has been optimized for low-abundance species.^31^ Synchronization was performed for MION and Orbitrap MS before creating a new data file, and the length of each file in this study was 10–60 min.

**Figure 1:**
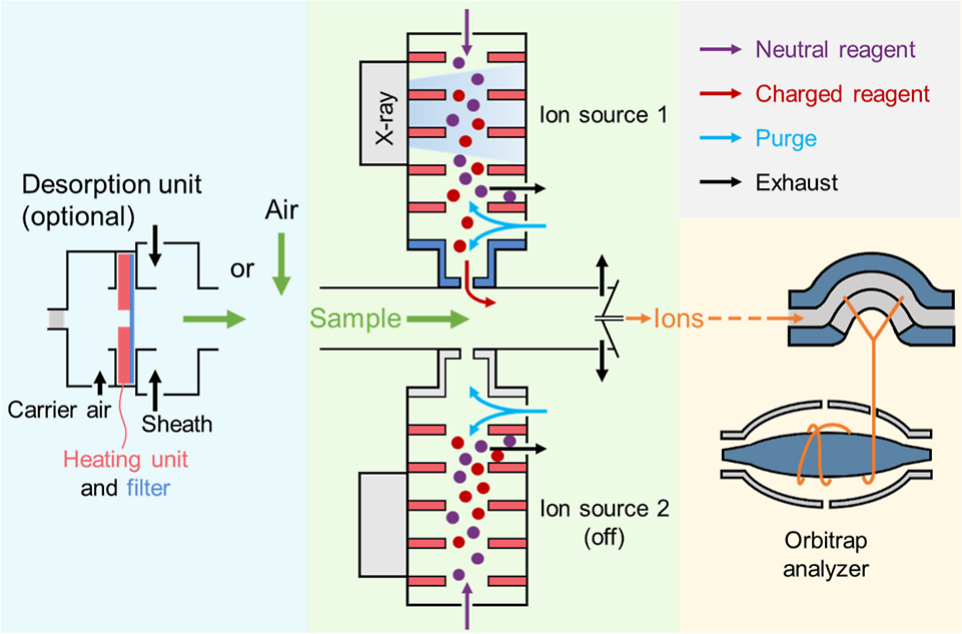
Illustrative schematic of the MION-Orbitrap. The blue, green, and yellow shaded areas indicate sampling, ionization, and mass spectrometric detection, respectively. Liquid samples can be vaporized using a thermal desorption unit, while gaseous samples can be directly measured at atmospheric pressure.

C_4_H_12_N^+^ and NO_3_^–^ were used as reagent ions with positive and negative polarities, respectively. The choice of reagent ions is mainly determined by the particular classes of trace species of interest. NO_3_^–^ is widely used in atmospheric studies for its high selectivity toward sulfuric acid and highly oxidized organic species.^2^ C_4_H_12_N^+^ can detect a broad range of organic species,^24^ and it has good sensitivity to Criegee intermediates.^20^ Dry purified air was passed slowly (5 mL min^–1^) through two separate vials containing liquid-phase diethylamine and nitric acid, feeding these reagent molecules to the corresponding reagent ion source of the MION inlet. As shown in Figure 1, the reagent molecules were ionized using a bipolar soft X-ray source (4.9 keV Hamamatsu L12536). A purge flow was applied to prevent the remaining electrically neutral reagent molecules from entering the sampling line by pushing them against the direction of the reagent flow, whereas charged reagent ions were accelerated by the electric field and then guided through an outlet orifice. A reagent ion source can be rapidly disabled by turning off X-ray radiation and setting the voltage on the outlet orifice to zero, facilitating the fast switching of the MION between the positive and negative ion sources.

**Scheme 1:**
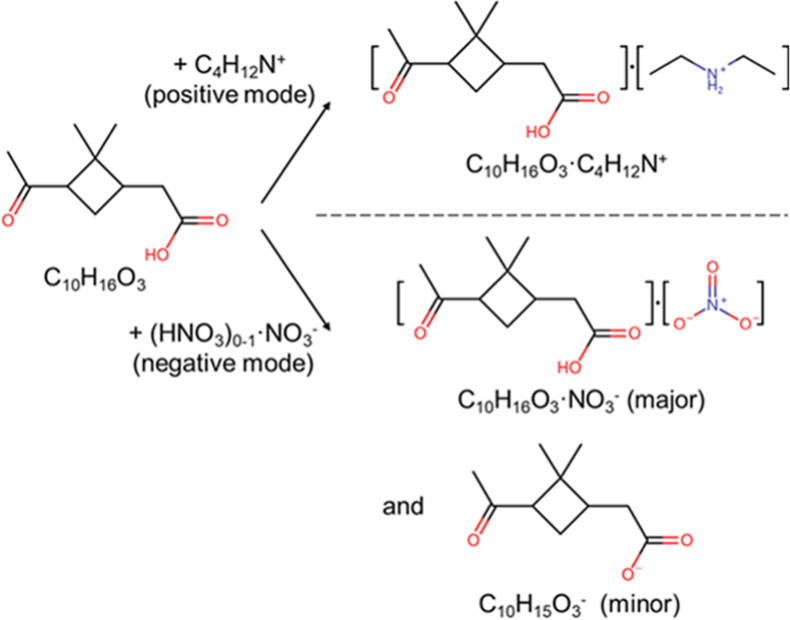
CI of Pinonic Acid (C_10_H_16_O_3_) in Both Polarities Nitrate ions can ionize pinonic acid, but we note that this efficiency is usually low.

A sample air flow carrying gaseous analytes entered the MION Orbitrap at a flow rate of 20 L min^–1^. The stainless-steel sampling tube was 1.2 m long, with an inner diameter of 23.5 mm. Analyte molecules were mixed with reagent ions at the end of the sampling line, experiencing a short ionization time (∼35 ms, estimated using average speed), and then entered the Orbitrap MS via a heated capillary (200 °C). The ionization reactions are illustrated using pinonic acid (an intermediate product of α-pinene ozonolysis) as an example (see Scheme 1).

In the positive mode, analytes were charged by C_4_H_12_N^+^, forming mostly C_4_H_12_N^+^-clustered ions. Analytes with high proton affinity, such as some Criegee intermediates, may also form protonated ions.^20^ In the negative mode, analytes were charged by NO_3_^–^ and HNO_3_·NO_3_^–^, forming NO_3_^–^-clustered ions or deprotonated ions. For the OOMs and the pesticides used in this study, NO_3_^–^-clustered ions are the main ionization products, while we also account for their corresponding deprotonated ions in the analysis. The C_4_H_12_N^+^- and NO_3_^–^-clustered ions might experience declustering and fragmentation inside the Orbitrap MS. We minimized this effect by tuning the voltage of the ion guides and limiting the number of ions accumulated for each Orbitrap injection.

The pesticide sample, provided by the Finnish Customs, was a standard mixture of 71 pesticides diluted in acetonitrile. The mass concentration of each pesticide was 0.1 mg kg^–1^. A full list of these pesticides is given in Table S1. We vaporized these pesticides using a custom-made thermal desorption unit (calibrator, Karsa Ltd.) and analyzed them using the polarity switching MION-Orbitrap instrument (Figure 1). The pesticide solution was injected onto a metal mesh filter using a 10 μL syringe (Hamilton). Pesticides vaporized from the heated filter were carried by a 1 L min^–1^ purified air flow, mixed with a 19 L min^–1^ sheath air flow, and then entered into the sampling line of the MION-Orbitrap. Detailed information on this thermal desorption unit can be found elsewhere.^7^

Reactive gaseous organic species were generated via α-pinene ozonolysis in a Teflon environmental chamber. These molecules, referred to as OOMs, represent typical oxidation products of biogenic volatile organic compounds in the atmosphere, which are important precursors for the formation of secondary organic aerosols.^34^ The volume of the environmental chamber was 2 m^3^, and the total flow rate entering the chamber was 45 L min^–1^. We flushed the chamber for more than 1 week before conducting experiments to minimize the influence of residues from previous experiments. α-Pinene was injected into a 1 L min^–1^ nitrogen flow using a 100 μL syringe (Hamilton) and a syringe pump (Chemyx Fusion 100) and then introduced to the chamber. Ozone was generated by an ozone generator (Dasibi 1008 PC), and its concentration in the chamber was ∼45 ppb before reacting with α-pinene. The maximum α-pinene concentration during the experiment was estimated to be ∼50 ppb. Detailed information on this environmental chamber has been reported previously.^24,35^

The raw mass spectra measured by the MION-Orbitrap were analyzed using Orbitool^36^ (version 2.5.1, last access date Dec. 13, 2023). After denoising and mass calibration, we identified pesticide peaks, assigned chemical formulas to the OOM peaks, and obtained their time series. We applied a signal-intensity-based correction to every peak to address the nonlinear sensitivity of the Orbitrap MS to trace species.^31^ We also performed sensitivity calibration for sulfuric acid^37^ and the mass-dependent transmission calbiration^38^ of the Orbitrap MS and derived the concentrations of OOMs from intensity-corrected signals using these calibration results.

## Results and Discussion

### Polarity Switching

The MION-Orbitrap can switch the measurement cycle between polarities within 1 min, analyzing trace species with high resolving power. The need for a switching frequency is associated with the rate of change of analyte concentrations, and it varies with specific applications. For instance, when trace species are analyzed in atmospheric chemistry studies, mass spectrometric data are usually averaged for 5–30 min to achieve a good signal-to-noise ratio. Accordingly, the MION-Orbitrap can switch polarities sufficiently fast to meet the needs of most atmospheric studies.

**Figure 2:**
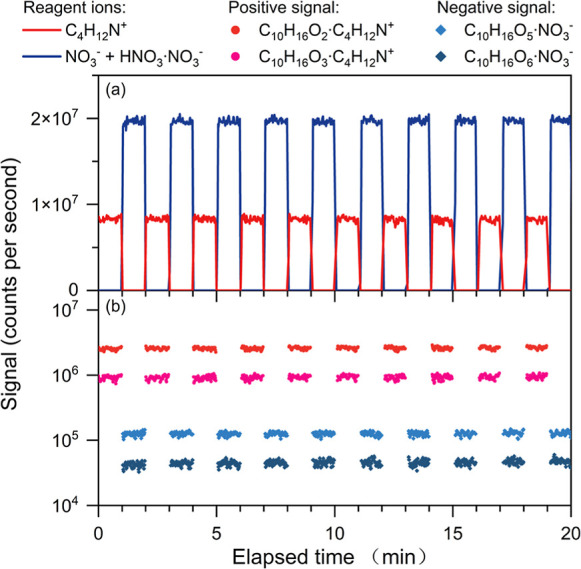
Time series of (a) reagent ions and (b) ionized OOMs measured by MION-Orbitrap with a 1 min polarity switching. The signals of ionized OOMs during polarity switching (1–2 scans), corresponding to the sharp changes in reagent ion signals, are not shown in panel (b).

Figure 2 shows an example of the chamber experiments. The MION-Orbitrap was operated with a measurement period of 1 min for each polarity. When the MION-Orbitrap was switched to, for instance, positive polarity, the C_4_H_12_N^+^ signal increased from 0 to a plateau within 1–2 scans (∼1–2 s), and the C_4_H_12_N^+^-clustered OOM signals appeared together with the C_4_H_12_N^+^ signal. The plateaus of the reagent ions and the OOMs were stable over time, showing that polarity switching had negligible interference with precision of signals. NO_3_^–^, HNO_3_·NO_3_^–^, and nitrate-ionized OOMs were by nature absent in the positive mode, which shows the advantage of polarity switching in minimizing the interference between reagent ions over switching ionization chemistries within the same polarity.

We also tested a faster 12 s switching for each polarity with the Orbitrap MS operated at a higher scan rate (auto gain control target = 5 × 10^4^) and observed stable signals over time (Figure S1). Higher switching frequency is arguably achievable with current settings or a lower resolving power, yet continuous measurements with this higher frequency require real-time synchronization between the software for the MION and the Orbitrap MS as an improvement over synchronizing only at the beginning of each data file. We note that using a high switching frequency may cause the loss of data as it takes 1–2 scans to switch the polarity. For this reason, we use a relatively low switching frequency (7.5 min of switching) for the chamber experiments. Nevertheless, the capability to switch and measure in 12 s already favors many applications, such as the fast screening of explosives and pesticide residues.

### Pesticide Detection

**Figure 3:**
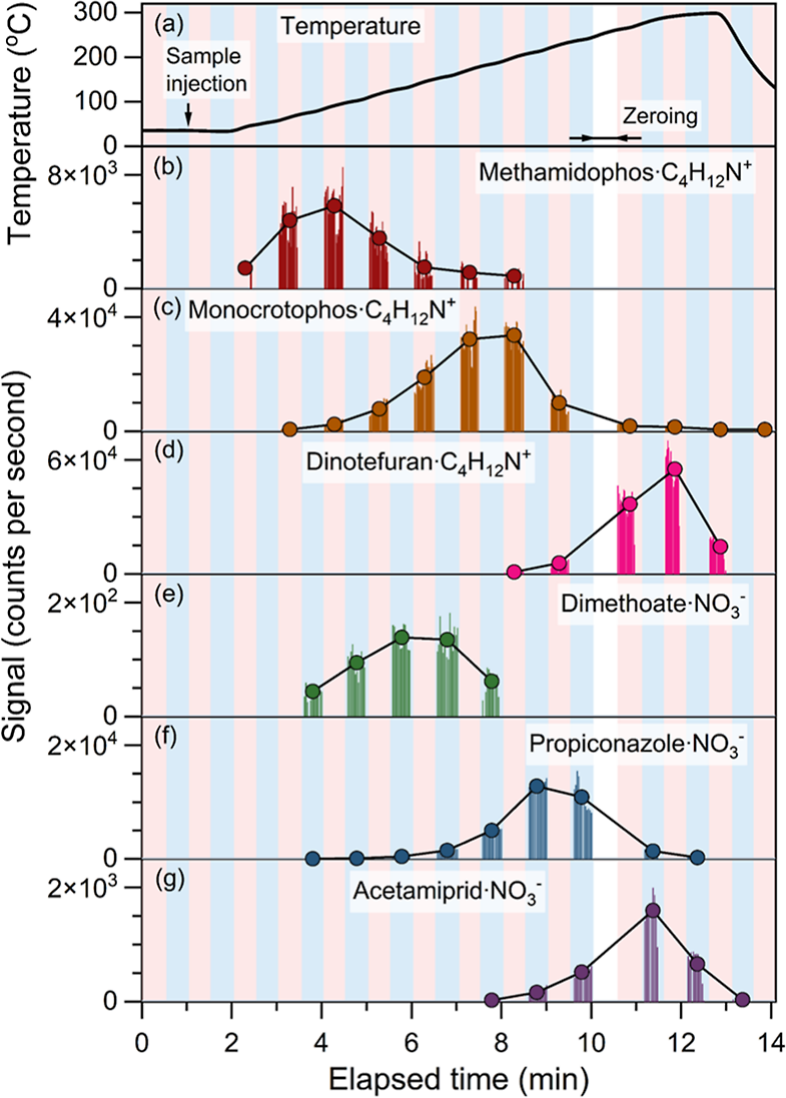
Time series of (a) temperature and (b–g) six pesticides during a thermal desorption experiment. The MION-Orbitrap was switched between positive (red) and negative (blue) polarities. Both reagent ion sources were turned off during the zeroing period to measure the background of the MION-Orbitrap.

The polarity-switching MION-Orbitrap captured the thermal desorption profile of pesticides well. The pesticide sample on the filter was gradually heated from 30 to 300 °C in 10 min. Figure 3 shows the time series of 6 pesticides. These pesticides were vaporized shortly after the beginning of the heating and unambiguously identified as C_4_H_12_N^+^- or NO_3_^–^-clustered ions. It also shows that the MION-Orbitrap could follow the variation of vaporized pesticides with 1 min switching. Using a higher switching frequency can obtain smoother desorption profiles or enable faster workflows with a higher temperature ramping rate.

We detected 23 of 71 pesticides using NO_3_^–^ as the reagent ion in the negative mode. The number of detected pesticides was 47 in the positive mode, as aminium is known to be sensitive to a broad range of species.^24^ Combining the results from both polarities, we detected 59 of the 71 pesticides (Table S1). The time series obtained from both polarities showed good consistency for the pesticides detected in both modes (Figure S2). Replacing NO_3_^–^ with less selective reagent ions such as bromide may benefit the detection;^7^ nevertheless, these results show that combining the results from both polarities can improve the rapid screening of pesticides by extending the range of detectable species. Also, polarity switching enables the analysis of pesticides using one instrument and one sample such that it helps to reduce costs, measurement time, and consumption of samples.

**Figure 4:**
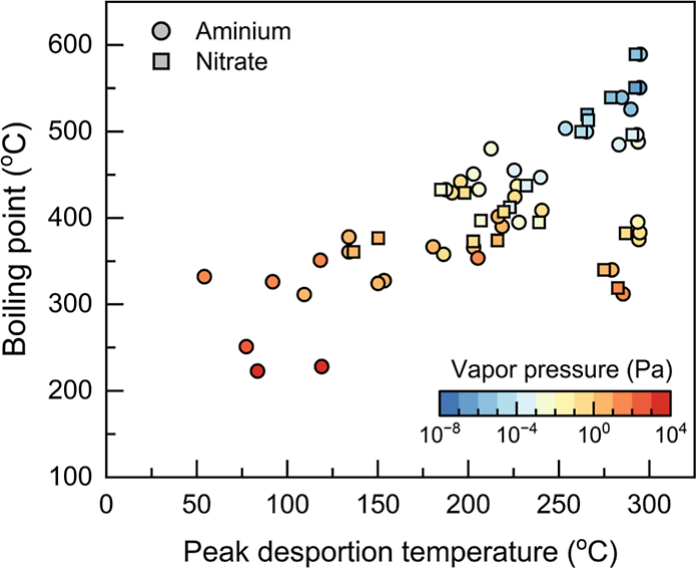
Boiling point and saturation vapor pressure of the detected pesticides as a function of the peak desorption temperature. The boiling point and saturation vapor pressure were predicted using MPBPWIN v1.42,^49^ and the peak desorption temperature was determined using the measured time series. Note that the uncertainties in the MPBPWIN predicted values affected their correlation with the peak desorption temperature.^50^

We correlated the measured peak desorption temperature with the predicted saturation vapor pressure and boiling point of the detected pesticides. Good correlations were observed despite the uncertainties in the predicted values (Figure 4), which are consistent with the fact that pesticides with a lower volatility need to be vaporized at higher temperatures. Previous studies have successfully used the peak desorption temperature to characterize the volatility of organic compounds,^39^ providing information on the composition and growth mechanisms of atmospheric aerosols.^40^ Here, we show that with 1 min or faster polarity switching, the MION-Orbitrap may aid thermal desorption analysis with extended detectability of trace species.

### OOMs Detection

**Figure 5:**
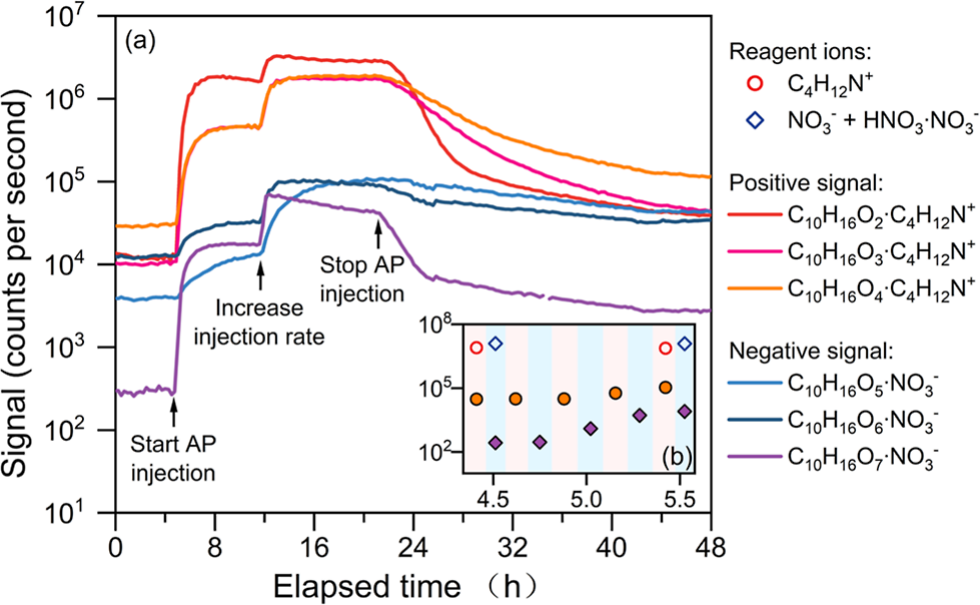
Time series of reagent ions and representative OOMs measured in a chamber experiment. These OOMs were generated by α-pinene (AP) ozonolysis. The ozone concentration reached a stable value of ∼45 ppb before the α-pinene injection. The MION-Orbitrap switched between the positive and negative polarities, as illustrated in panel (b). Filled markers in panel (b) show the average signals of C_10_H_16_O_4_·C_4_H_12_N^+^ and C_10_H_16_O_7_·NO_3_^–^ during each switching cycle, and they share the same axes as those in panel (a).

We further investigated the capability of the polarity switching MION-Orbitrap instrument to detect gaseous OOMs. Figure 5a shows the time series of C_10_H_16_O_2–7_ OOMs, which are typical oxidation products of α-pinene (C_10_H_16_). The ozone concentration and background signals of OOMs were stable at the beginning of the experiment. α-Pinene was injected into the chamber at ∼5 h (elapsed time), where it reacted with ozone and produced OOMs rapidly,^41^ leading to a sharp increase in OOM signals. We increased the injection rate of α-pinene at ∼13 h and stopped the injection at ∼21 h, and the detected OOM signals varied correspondingly. Among the C_10_H_16_O_2–7_ OOMs shown in Figure 5a, C_10_H_16_O_2_ and C_10_H_16_O_7_ responded faster to α-pinene injection than others, especially when the injection was stopped. This is consistent with the understanding that the buffering effect due to gas-wall partitioning mainly influences species with moderate-volatility species that exist in both phases with comparable concentrations^42,43^ as high-volatility species mainly exist in the gas phase, and the evaporation of low-volatility species affects their gas-phase concentrations minorly due to the low saturation vapor pressure. We also note that aerosol production was not intentionally suppressed in this experiment, which also affected the variation of OOM concentrations according to our previous experiments.^9^

By switching the polarity of the MION-Orbitrap 8 times per hour (Figure 5b), we obtained a smooth time series of the OOMs. This shows that the on average 7.5 min switching followed the temporal evolution of OOMs with good stability over the 2 day continuous chamber experiment. Such a switching frequency was determined according to the typical averaging time (5–30 min) for mass spectrometric data from atmospheric measurements. Therefore, it is reasonable to conclude that the polarity switching MION-Orbitrap is applicable to online analysis of atmospheric OOMs with sufficient switching frequency.

**Figure 6:**
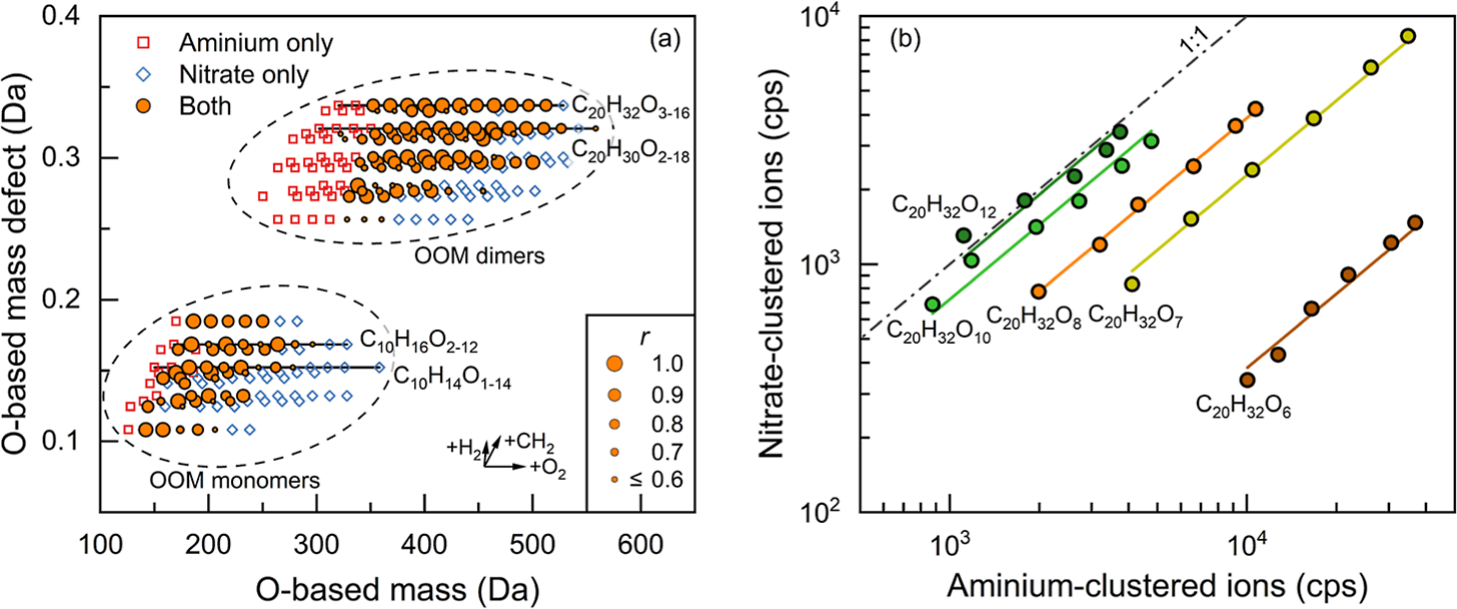
Correlations of OOM signals measured in the chamber experiment between the positive and negative modes. (a) Kendrick-type diagram of C_7–10_ and C_16–20_ OOMs. The mass is herein defined based on O, i.e., the mass of an oxygen atom is defined as 16 Da. The horizontal and vertical axes show the exact O-based masses and mass defects of neutral molecules, with reagent ions subtracted from the measured formulas. The arrows indicate the change of the O-based mass and mass defect upon adding certain atoms. The size of the circle markers indicates the correlation coefficient (*r*) between the signals measured in the positive and negative modes. (b) Signals of C_20_ OOMs measured in the positive and negative modes. The solid lines are the trend lines of the measured data on the logarithmic scale.

The C_4_H_12_N^+^ and NO_3_^–^ ionization chemistries are complementary to each other in terms of detecting the presence of OOMs with a wide range of oxidation states. Figure 6a presents the detected OOMs during α-pinene injection using an O-based mass defect. The mass is herein defined such that an O atom is 16 Da, as the oxidization processes mainly add O atoms to OOMs, accompanied by the changes in the number of C and H atoms. To minimize the influence of the chamber background, we limited our analysis to C_7–10_ and C_16–20_ species whose concentration increased significantly during the α-pinene injection. These compounds are referred to as OOM monomers and dimers, respectively.^3^ NO_3_^–^ is known to be selective to highly oxygenated organic species,^24^ consistent with the OOM monomers and dimers detected in the negative mode. With a broad detectability, C_4_H_12_N^+^ in general shows a preference for OOMs with low oxidation states, which is slightly different from previous findings.^24^ This preference is probably associated with the sensitivity of C_4_H_12_N^+^ to different functional groups. Analogous to ionization by ammonium,^44^ C_4_H_12_N^+^ may be highly sensitive to ketones. Further oxidation of an OOM produces multiple hydroperoxide functional groups,^3^ which can form internal hydrogen bonds and may weaken intramolecular interactions^45^ including C_4_H_12_N^+^-adduct ionization.

For the OOMs ionized by both NO_3_^–^ and C_4_H_12_N^+^, we observed good correlations between the signals detected in positive and negative polarities, while the correlations for dimers were on average better than those for monomers. For instance, extremely good correlations (*r* > 0.98) were observed for C_20_H_32_O_6–12_ OOM dimers (Figure 6b). However, relatively low (*r* < 0.6) correlations were found for some of the OOM monomers (Figures 6a and S3), reflecting the difference in the sensitivity of ionization chemistries to isomers formed by different oxidation processes.

There is concern about the broad sensitivity of the C_4_H_12_N^+^ ionization chemistry for complex samples. Being sensitive to a broad range of species, reagent ions can be readily scavenged, potentially affecting the accuracy of quantification. We observed such a depletion of C_4_H_12_N^+^ in chamber experiments for α-pinene ozonolysis. The C_4_H_12_N^+^ signal was >90% of the total signal at the beginning of the chamber experiments, and it dropped to ∼50% after the injection of ∼50 ppb α-pinene. Despite this, we saw good correlations between the signals measured in the positive and negative modes for the OOM dimers and some of the OOM monomers (Figure 6), indicating that the reduced reagent ion signal had only a minor effect on the quantification of the OOMs in these well-controlled chamber experiments with dry air.

**Figure 7:**
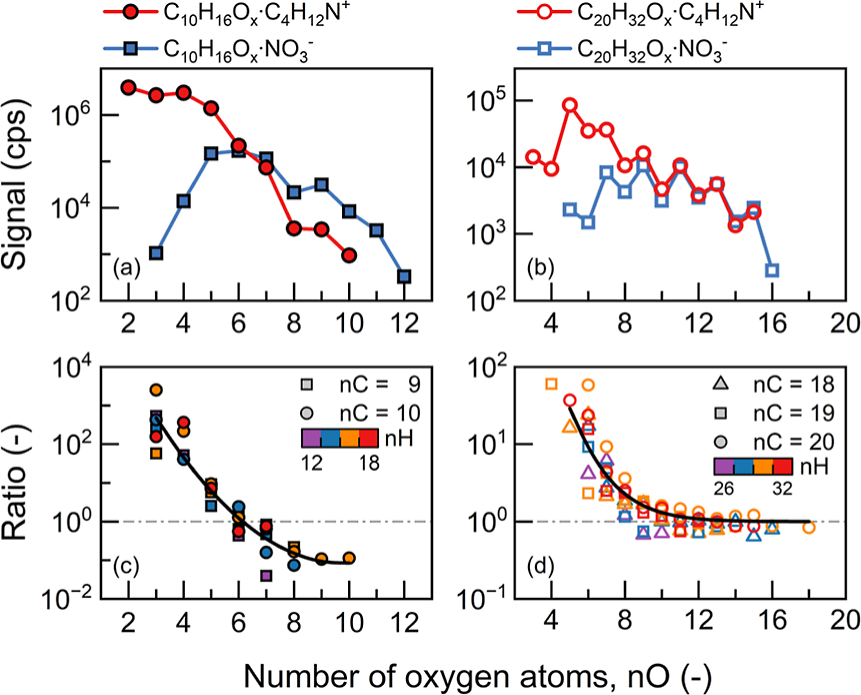
Signals of C_9–10_ and C_18–20_ OOMs (monomers and dimers, respectively) measured in the positive and negative modes as a function of the number of oxygen atoms (nO). (a) Signal of C_10_H_16_O_2–12_ monomers. (b) Signal of C_20_H_32_O_3–16_ dimers. (c) Ratio of monomer signals measured in the positive mode to those measured in the negative mode. The shape and color of markers indicate the number of carbon and hydrogen atoms (nC and nH), respectively. The solid line is the average ratio of the positive signal to the negative signal as a function of nO. The ratio of signals mainly reflects the ratio of sensitivities, though the former is also influenced by the reagent ion concentrations, which are 4 × 10^7^ and 1.5 × 10^7^ in the positive and negative modes, respectively. (d) Ratio of dimer signals measured in the positive mode to those in the negative mode.

We further quantified the sensitivity of NO_3_^–^ and C_4_H_12_N^+^ to the presence of the OOM monomers and dimers. The ratio of positive signals to negative signals had a strong dependence on the number of constituent oxygen atoms, though we note that the number of oxygen atoms was not the only determining factor of sensitivity. The signals of NO_3_^–^-clustered and C_4_H_12_N^+^-clustered ions were comparable for the ion-bound OOM dimers containing >10 oxygen atoms (Figure 7). Such a near one-to-one ratio between NO_3_^–^-clustered and C_4_H_12_N^+^-clustered ion signals may be associated with coincidences, as the measured signals were affected by the reagent ion concentrations and transmissions. Nevertheless, comparable dimer signals were also observed in a previous study using two separate instruments.^24^ Hence, we infer that the ionization of these highly oxygenated dimers by C_4_H_12_N^+^ was close to the theoretical maximum, as the ionization of these dimers by NO_3_^–^ can be approximated by a theoretical maximum determined by the collision rate between NO_3_^–^ and OOMs.^34^ For OOM dimers containing <10 oxygen atoms, C_4_H_12_N^+^ ionization was significantly more efficient than NO_3_^–^. In terms of OOM monomers, C_4_H_12_N^+^ was more sensitive to those containing <6 oxygen atoms and NO_3_^–^ was more sensitive to >7 oxygen atoms.

**Figure 8:**
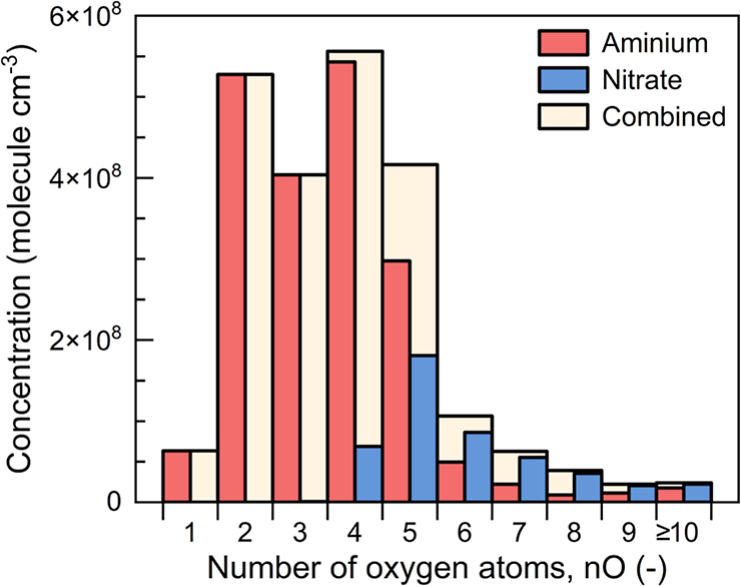
Concentrations of OOMs measured in positive and negative modes. The combined concentrations are calculated based on every identified chemical species, i.e., the higher concentration is used if a species is detected in both modes.

The results from the chamber experiments suggest that the polarity switching MION-Orbitrap is a promising tool to improve our understanding of the role of the OOMs in atmospheric environments. As summarized in Figure 8, only a minor fraction of OOMs were measured with the NO_3_^–^ ionization compared to the combined results from NO_3_^–^ and C_4_H_12_N^+^ ionization. The fraction of the OOMs measured in the positive and negative modes was 88 and 21% of the combined results, respectively. Due to the high selectivity of NO_3_^–^ toward high oxidation states, the OOMs detected with NO_3_^–^ tend to have high oxidation states and accordingly low volatility,^23^ being important to the nucleation and initial growth of new particles down to molecular cluster sizes.^12,13^ However, OOMs with moderate and low oxidation states can dominate the formation of large secondary organic aerosols^46^ as the OOM concentration decreases with an increasing oxidation state (e.g., Figure 7). They may also have low volatility in low-temperature environments, such as in winter or the upper troposphere, overcoming the Kelvin effect and being important to small particles.^25^Figure 8 indicates that combining results from NO_3_^–^ and C_4_H_12_N^+^ ionization can substantially improve the detection of OOMs with low oxidation states and address the need to compensate for the underestimation of OOMs with low oxidation states in atmospheric measurements using nitrate-CIMS. This can be especially important for polluted environments where the formation of high oxidation-state OOMs is suppressed by a high concentration of NO_*x*_.^47^ For instance, OOMs measured using nitrate-CIMS in polluted megacities mainly contain less than 8 constituent oxygen atoms.^48^ Accordingly, the polarity switching MION-Orbitrap has a large potential to improve the knowledge of OOMs by extending detection to a broader range of oxidation states.

## Conclusions

We have shown that combining a polarity switching mass spectrometer and a switching selective CI technique can enable the fast, separation-free, and sensitive analysis of trace species in complex samples. Using a polarity-switching MION Orbitrap, we could effectively detect and resolve a wide range of trace species by combining complementary ionization chemistries from both polarities. The choice of ionization chemistries depends on the particular classes of trace species of interest, and here, we used C_4_H_12_N^+^ and NO_3_^–^ as reagent ions. The detection and stability of the polarity switching MION-Orbitrap were tested by using a standard pesticide sample and reactive gaseous organic species generated by α-pinene ozonolysis.

The MION-Orbitrap can achieve fast polarity switching and stable measurements within 1 min (down to 12 s with synchronization at the beginning of each file), while its high resolving power and low detection limit facilitate the analysis of trace species. Combining results from both polarities, we obtained a broader range of detection than that using only one ionization chemistry. We detected 59 out of 71 pesticides in the standard pesticide sample: 23 were detected in the negative mode, and 47 were detected in the positive mode. The 1 min polarity switching captured the thermal desorption profiles of pesticides well, providing a good association between the peak desorption temperature and the saturation vapor pressure. For α-pinene ozonolysis products generated in an environmental chamber, NO_3_^–^ was selective to species with high oxidation states. C_4_H_12_N^+^ had a broader detectability, and it was, in general, more sensitive to species with low oxidation states. Accordingly, the polarity switching MION-Orbitrap with NO_3_^–^ and C_4_H_12_N^+^ could effectively detect reactive organic species ranging from a low oxidation state to the highest oxidation state. Other reagent ions, such as bromide, can also be used for the better detection of particular classes of analytes, e.g., halogen species.

The substantially extended detectability makes the MION-Orbitrap a promisingly powerful tool for online nontargeted analysis. Compared to online CIMSs using one ionization chemistry, the polarity switching MION-Orbitrap can uncover a more comprehensive picture of trace species in a complex sample, aiding analysis in various applications without compromising versatility.

## Supporting Information Available

It includes the time series of α-pinene ozonolysis products measured with 12 s polarity switching, time series of methoxyfenozide during a thermal desorption experiment, simplified chemical reaction scheme of peroxy radicals in α-pinene ozonolysis, time series of OOMs with different correlation coefficients between positive and negative polarities, mass defects of OOMs detected in both polarities, and list of pesticides in the standard sample (PDF)

## Notes

Joona M., Jyri M., P.J., A.S., and F.P. work for Karsa. DW works for Aerodyne.
