# Supplementary material for: Extending the Range of Detectable Trace Species with the Fast Polarity Switching of Chemical Ionization Orbitrap Mass Spectrometry
Source: Anal Chem. 2024 May 1;96(21):8604–12. doi: 10.1021/acs.analchem.4c00650 (PMC11393793; doi:10.1021/acs.analchem.4c00650)
Supplement: Supplementary file 1 — ac4c00650_si_001.pdf [file ac4c00650_si_001.pdf]

Supporting Information

Extending the range of detectable trace species with fast polarity switching of chemical ionization Orbitrap mass spectrometry

Runlong Cai[a,b,*], Joona Mikkilä[c], Anna Bengs[b], Mrisha Koirala[b], Jyri Mikkilä[c], Sebastian Holm[b], Paxton Juuti[c], Melissa Meder[b], Fariba Partovi[c,d], Aleksei Shcherbinin[c], Douglas Worsnop[b,d], Mikael Ehn[b], Juha Kangasluoma[b,c]

[a]Shanghai Key Laboratory of Atmospheric Particle Pollution and Prevention (LAP[3]), Department of Environmental Science & Engineering, Fudan University, 200438 Shanghai, China

[b]Institute for Atmospheric and Earth System Research/Physics, Faculty of Science, University of Helsinki, 00014 Helsinki, Finland

[c]Karsa Ltd., A. I. Virtasen aukio 1, 00560 Helsinki, Finland

[d]Faculty of Engineering and Natural Sciences, Tampere University, 33720 Tampere, Finland

[*]Correspondence to Runlong Cai, runlong_cai@fudan.edu.cn

Table of Contents

Figure S1. 2
Figure S2. 2
Scheme S1. 3
Figure S3. 3
Figure S4. 4
Table S1. 4

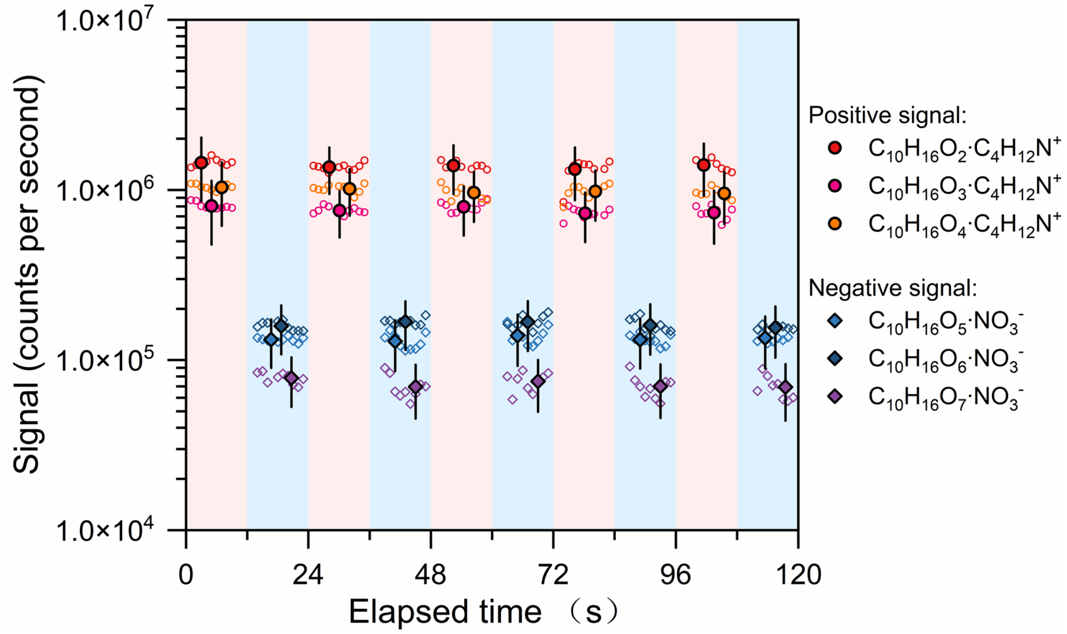

Figure S1. $C_{10}H_{16}O_{2-7}$ molecules measured by the MION-Orbitrap with 12-s polarity switching. The shaded background in red and blue indicates the positive and negative modes, respectively. Open markers show the signals in every scan. Closed markers and the error bars represent the mean values and standard deviations, respectively. The closed markers are shifted horizontally to avoid overlapping.

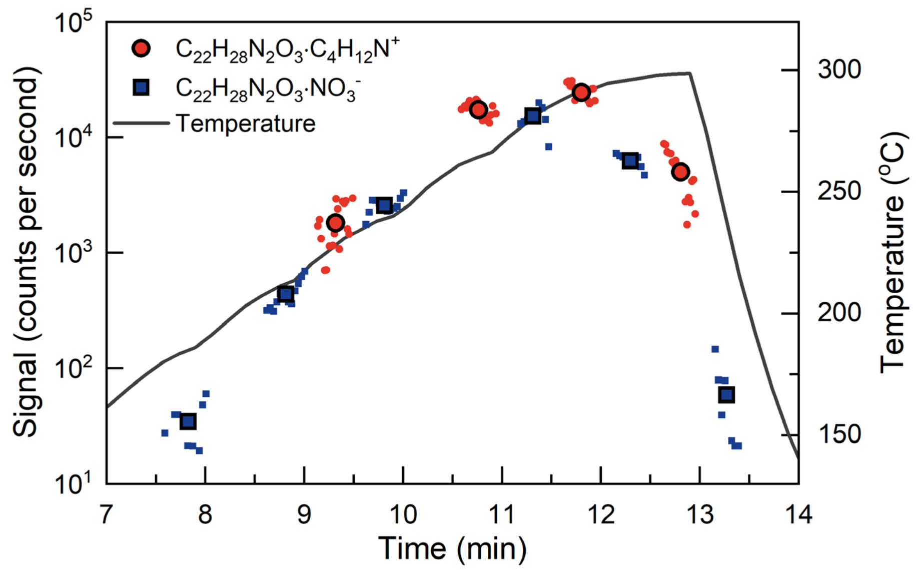

Figure S2. Time series of methoxyfenozide ($C_{22}H_{28}N_2O_3$) during a thermal desorption experiment. $C_4H_{12}N^+$- and NO_3^--clustered methoxyfenozide signals were detected in the positive and negative modes, respectively. The small markers indicate the results from every scan and the large markers indicate the average value of every switching cycle. Note that the sensitivity of MION-Orbitrap varies with the ionization chemistry, hence the absolute signals obtained from both polarities are not directly comparable. This figure shows that the thermal desorption profiles measured in both polarities were consistent with each other.

$RO_2\cdot \rightarrow RO_4\cdot \rightarrow RO_4\cdot \rightarrow \cdots$	Autoxidation	(S1)
$RO_2\cdot \rightarrow R(=O) + HO\cdot$	Unimolecular termination via H-shift	(S2)
$RO_2\cdot + R'O_2\cdot \rightarrow R\text{-}OH + R'(=O) + OH$	Carbonyl and hydroxyl formation	(S3)
$RO_2\cdot + R'O_2\cdot \rightarrow RO\cdot + R'O\cdot + O_2$	Formation of alkoxy radicals	(S4)
$RO_2\cdot + R'O_2\cdot \rightarrow ROOR' + O_2$	OOM dimer formation	(S5)
$RO_2\cdot + HO_2\cdot \rightarrow RO\cdot + HO\cdot + O_2$	Alkoxy radical and OH radical formation	(S6)
$RO_2\cdot + HO_2\cdot \rightarrow ROOH + O_2$	Hydroperoxide formation	(S7)

Scheme S1. Simplified chemical reaction scheme of peroxy radicals in α-pinene ozonolysis.

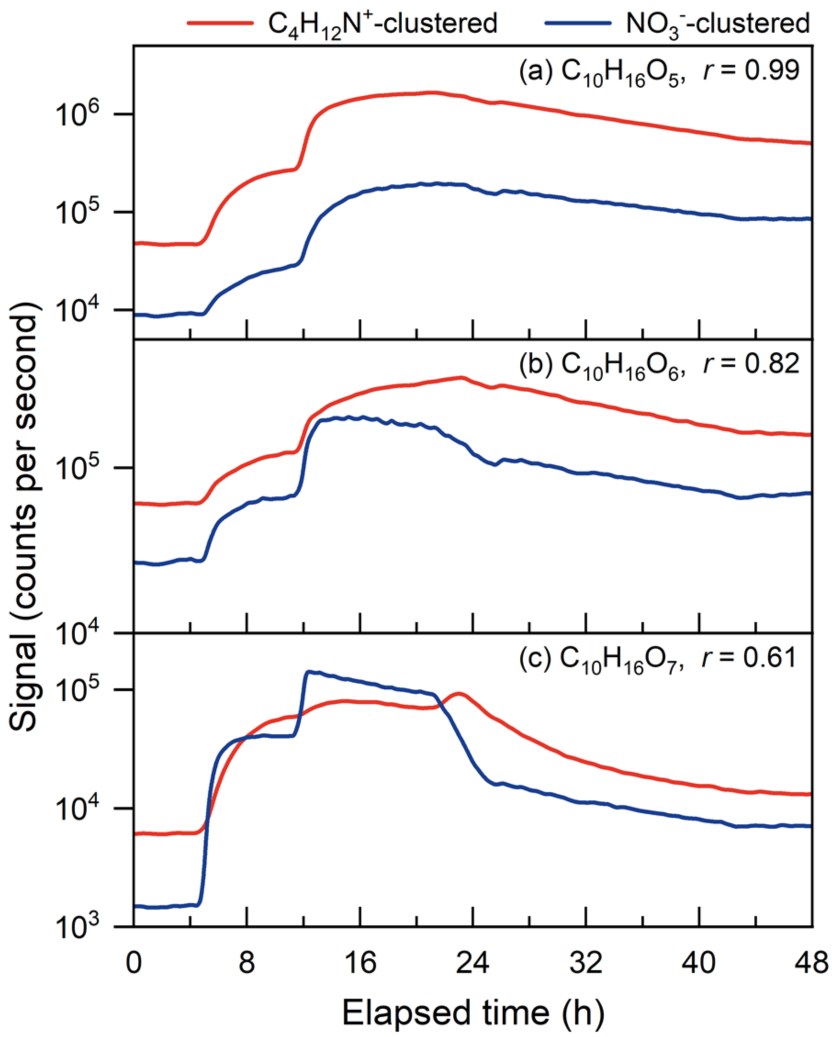

Figure S3. Examples of oxygenated organic molecule signals measured in the chamber experiment with different correlation coefficients (r) between the positive and negative polarities. The correlation coefficient was calculated using signals in the logarithmic scale between 6 and 30 h.

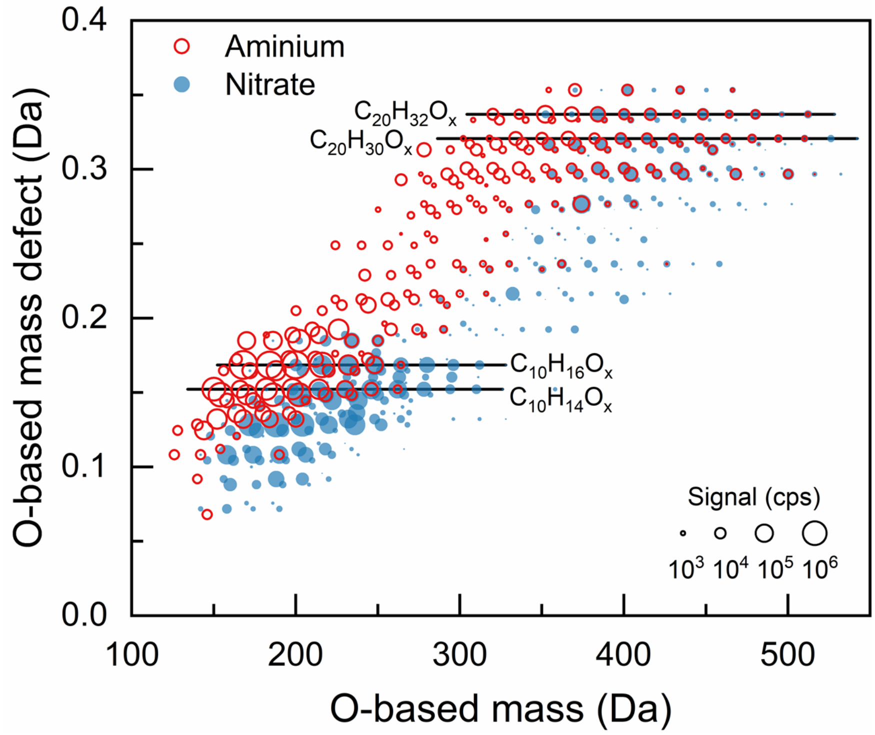

Figure S4. Kendrick-type diagram of neutral oxygenated organic molecules measured in the α-pinene ozonolysis chamber experiment. Signal intensity is indicated by the marker size. The mass is defined based on O, i.e., adding one or a few oxygen atoms to a certain formula does not change the O-based mass defect. All the molecules shown in this figure have significant positive correlations with α-pinene ozonolysis injection; however, we note that a few of these molecules may not be formed via pure α-pinene ozonolysis due to the influence of chamber background.

Table S1. Complete list of pesticides in the standard sample and their detection by aminium and nitrate chemical ionization.

Name	Formula	Detected with aminium	Detected with nitrate
Methamidophos	$C_2H_8NO_2PS$	True	
Methomyl	$C_5H_{10}N_2O_2S$	True	
Propamocarb	$C_9H_{20}N_2O_2$		
Aldicarb	$C_7H_{14}N_2O_2S$	True	
Carbendazim	$C_9H_9N_3O_2$		
Pyrimethanil	$C_{12}H_{13}N_3$		
Thiabendazole	$C_{10}H_7N_3S$		
Carbaryl	$C_{12}H_{11}NO_2$	True	
Dinotefuran	$C_7H_{14}N_4O_3$	True	
Carbofuran	$C_{12}H_{15}NO_3$	True	
Acetamiprid	$C_{10}ClH_{11}N_4$	True	True
Monocrotophos	$C_7H_{14}NO_5P$	True	
Mepanipyrim	$C_{14}H_{13}N_3$	True	
Ethiofencarb	$C_{11}H_{15}NO_2S$	True	
Methiocarb	$C_{11}H_{15}NO_2S$	True	
Dimethoate	$C_5H_{12}NO_3PS_2$	True	True

Flonicamid	$C_9F_3H_6N_3O$		True
Pirimicarb	$C_{11}H_{18}N_4O_2$	True	
Oxydemeton-methyl	$C_6H_{15}O_4PS_2$	True	
Linuron	$C_9Cl_2H_{10}N_2O_2$	True	
Thiacloprid	$C_{10}ClH_9N_4S$	True	
Propyzamide	$C_{12}Cl_2H_{11}NO$		True
Imidacloprid	$C_9ClH_{10}N_5O_2$	True	
Phorate	$C_7H_{17}O_2PS_3$		True
Disulfoton	$C_8H_{19}O_2PS_3$	True	True
Fenthion	$C_{10}H_{15}O_3PS_2$		
Oxadixyl	$C_{14}H_{18}N_2O_4$	True	
Metalaxyl	$C_{15}H_{21}NO_4$	True	
Terbufos	$C_9H_{21}O_2PS_3$	True	
Myclobutanil	$C_{15}ClH_{17}N_4$		True
Thiamethoxam	$C_8ClH_{10}N_5O_3S$	True	
Triadimefon	$C_{14}ClH_{16}N_3O_2$		True
Amitraz	$C_{19}H_{23}N_3$		
Imazalil	$C_{14}Cl_2H_{14}N_2O$		
Fenhexamid	$C_{14}Cl_2H_{17}NO_2$	True	
Flutriafol	$C_{16}F_2H_{13}N_3O$		True
Fenamiphos	$C_{13}H_{22}NO_3PS$	True	
Buprofezin	$C_{16}H_{23}N_3OS$	True	
Quinoxyfen	$C_{15}Cl_2FH_8NO$		
Tebuconazol	$C_{16}ClH_{22}N_3O$	True	True
Fensulfothion	$C_{11}H_{17}O_4PS_2$	True	
Diflubenzuron	$C_{14}ClF_2H_9N_2O_2$	True	True
Flusilazole	$C_{16}F_2H_{15}N_3Si$		True
Bupirimate	$C_{13}H_{24}N_4O_3S$	True	True
Phosmet	$C_{11}H_{12}NO_4PS_2$	True	
Penflufen	$C_{18}FH_{24}N_3O$	True	
Pyriproxyfen	$C_{20}H_{19}NO_3$	True	
Diniconazole	$C_{15}Cl_2H_{17}N_3O$		True
Fenarimol	$C_{17}Cl_2H_{12}N_2O$		True
Malathion	$C_{10}H_{19}O_6PS_2$	True	
Fenpyrazamine	$C_{17}H_{21}N_3O_2S$	True	
Propiconazole	$C_{15}Cl_2H_{17}N_3O_2$		
Boscalid	$C_{18}Cl_2H_{12}N_2O$		True
Propargite	$C_{19}H_{26}O_4S$	True	
Hexythiazox	$C_{17}ClH_{21}N_2O_2S$		
Methoxyfenozide	$C_{22}H_{28}N_2O_3$	True	True
Spirotetramat	$C_{21}H_{27}NO_5$	True	
Famoxadone	$C_{22}H_{18}N_2O_4$	True	True
Teflubenzuron	$C_{14}Cl_2F_4H_6N_2O_2$		True
Fluopicolide	$C_{14}Cl_3F_3H_8N_2O$	True	
Pyraclostrobin	$C_{19}ClH_{18}N_3O_4$	True	
Dimethomorph	$C_{21}ClH_{22}NO_4$	True	True
Fluopyram	$C_{16}ClF_6H_{11}N_2O$	True	True
Difenoconazole	$C_{19}Cl_2H_{17}N_3O_3$	True	
Trifloxystrobin	$C_{20}F_3H_{19}N_2O_4$	True	
Mandipropamid	$C_{23}ClH_{22}NO_4$	True	True
Chlorantraniliprole	$BrC_{18}Cl_2H_{14}N_5O_2$		
Flufenoxuron	$C_{21}ClF_6H_{11}N_2O_3$	True	
Lufenuron	$C_{17}Cl_2F_8H_8N_2O_3$		True
Indoxacarb	$C_{22}ClF_3H_{17}N_3O_7$	True	
Flubendiamide	$C_{23}F_7H_{22}IN_2O_4S$		

Note: pesticides were detected as aminium- and nitrate-clustered ions in the positive and negative modes, respectively, except for teflubenzuron, which was detected as deprotonated ions in the negative mode.
